# Editorial: New insights in sarcoidosis: from bench to bedside

**DOI:** 10.3389/fmed.2023.1202435

**Published:** 2023-05-04

**Authors:** Paolo Cameli, Davide Biondini, Alfonso Carleo, Carmel J. W. Stock

**Affiliations:** ^1^Respiratory Diseases Unit, Department of Medicine, Surgery and Neurosciences, University of Siena, Siena, Italy; ^2^Respiratory Disease Unit, Department of Cardiac, Thoracic, Vascular Sciences and Public Health, University of Padova, Padova, Italy; ^3^Department of Pneumology, Medical School Hannover (MHH), Hannover, Germany; ^4^Interstitial Lung Disease Unit, Royal Brompton and Harefield Clinical Group, Guy's and St Thomas' NHS Foundation Trust, London, United Kingdom

**Keywords:** sarcoidosis, multidisciplinary approach, omic, patients reported outcome measures, precision medicine

Sarcoidosis may be considered as an archetype of complex and multifactorial diseases, a disorder that results from a combination of multiple genetic and epigenetic variants or modifications associated with the influence of environmental, physiological, and pathological factors (1). Moreover, the knowledge gaps in sarcoidosis are still significant nowadays: reliable and widely accepted data on etiology, epidemiology, and pathogenesis are lacking, as well as a standardized phenotypic assessment of the disease that is able to reflect its clinical heterogeneity. The etiopathogenesis of sarcoidosis is far from fully understood; as a result, no validated pre-clinical, *in-vitro*, or animal models of the disease are available. As things stand, from a clinical point of view, there is no standardized approach for diagnosis, severity assessment, prognostic estimation, or pharmacological and non-pharmacological treatment. Although international guidelines on the diagnosis and treatment of sarcoidosis have been recently released, in both documents it was clearly stated that higher quality evidence is still urgently needed to allow recommendations with a higher level of confidence and evidence of efficacy (2, 3).

Based on this framework, the current Research Topic contributes to these issues through the publication of articles focused on innovative and very different aspects of sarcoidosis. The role of genomic analysis in sarcoidosis is attracting growing interest considering that the interplay between genetic and environmental factors is a key step in disease immunopathology. The article by Al Hayja et al. focused on providing a better definition of the interplay between already described genetic variants from the HLA locus and the cellular expression and percentage of bronchoalveolar lavage (BAL) in a large cohort of patients, stratified according their clinical presentation: Lofgren and non-Lofgren. The results provided evidence that BAL cellular expression was directly driven by genomic expression, further highlighting the crucial role of HLA polymorphisms not only in determining the susceptibility to sarcoidosis but also in defining the clinical phenotype and endotype of the disease from the onset. In this field, the implementation of next-generation sequencing for HLA typing was conducted by Sikorova et al. to confirm the association between already described HLA polymorphisms and specific phenotypes of the disease (4), as well as the identification of new potential HLA variants that may be correlated to different clinical expressions and outcomes, expanding the knowledge of the influence of HLA on sarcoidosis immunopathogenesis. Moreover, the article by Casanova et al. provided interesting insights into the interplay between genetic architecture and the clinical course of sarcoidosis: new biomarkers were investigated both in plasma and lung and lymph node tissues. The results showed that HBEGF was more expressed in patients with complicated disease, while eNAMPT and ANG-2 could possibly predict the development of sarcoid-induced lung fibrosis, which represents one of the most threatening and potentially lethal expressions of sarcoidosis. The scientific soundness of these findings was further enhanced by genomic analysis, which confirmed the correlation between biomarkers' plasma and tissue concentrations and dysregulation of the specific protein-coding gene expression.

In a bench-to-bedside approach, clinical research is crucial to boost the accuracy of diagnosis and therapeutic management of sarcoidosis, especially considering the nature of the great mimicker disease, expressed both in terms of multi-systemic localization and heterogeneous characterization of extrapulmonary sarcoid lesions. In this field, the contributions of Boch et al. and Lečić et al. are surely of interest, since they are focused on better defining specific issues that may represent relevant dilemmas in clinical practice: ultra-rare localizations of the disease (like thyroid) and heterogeneous expression of a well-recognized site of interest of sarcoidosis, such as the skin. Besides providing an extensive description, these studies further underscored the importance of a multidisciplinary approach in patients with sarcoidosis, that must be implemented by specialists with sarcoidosis-targeted expertise in order to achieve satisfying outcomes, as described by the latest guidelines (2, 3). The article by Caffarelli et al. emphasized this specific Research Topic, focusing on the dysregulations of calcium metabolism and bone fragility risk in patients with sarcoidosis. Even though bone localizations and hypercalcemia plus hypercalciuria are well-known complications of sarcoidosis (5), the authors implied that disease severity and activity could represent the main risk factors for bone fragility, superior to the use of steroid treatment. Considering that osteoporosis and bone fractures represent the most common causes of disability and morbidity worldwide, these findings further highlighted the potential impact of sarcoidosis on quality of life and supported the urgent need to validate multidimensional patients-reported outcomes measures (PROMs), to be eventually implemented in the future clinical trials. In this regard, the article by Hofauer et al. investigated the clinical course of sicca syndrome, which represents a potentially disabling symptom in patients with sarcoidosis, in a pluriannual follow-up, showing that only a small percentage achieved good symptom control and that the therapies used did not show any evidence of efficacy on this issue.

In conclusion, the present Research Topic describes the hottest topics in sarcoidosis research (the implementation of omics, multidisciplinary approaches, and PROMs), reporting innovative and intriguing insights for a better comprehension of disease pathogenesis and for the optimization of clinical assistance.

## Author contributions

PC: conceptualization and writing of the editorial. DB, AC, and CS: supervision and revision. All authors contributed to the article and approved the submitted version.
